# Clinical study of Mito-FLAG regimen in treatment of relapsed acute myeloid leukemia

**DOI:** 10.3892/etm.2013.917

**Published:** 2013-01-22

**Authors:** SHENG LUO, FANGFANG CAI, LEI JIANG, SHENGHUI ZHANG, ZHIJIAN SHEN, LAN SUN, SHENMENG GAO

**Affiliations:** 1 Department of Hematology, The First Affiliated Hospital of Wenzhou Medical College, Wenzhou, Zhejiang 325000, P.R. China; 2 Laboratory of Internal Medicine, The First Affiliated Hospital of Wenzhou Medical College, Wenzhou, Zhejiang 325000, P.R. China

**Keywords:** fludarabine, Mito-FLAG, relapsed acute myeloid leukemia, salvage chemotherapy

## Abstract

Patients with relapsed acute myeloid leukemia (AML) have unfavorable prognosis and require innovative therapeutic approaches. In this study we used fludarabine combined with a middle dose of cytosine arabinoside (Ara-C), mitoxantrone and granulocyte-colony stimulating factor (G-CSF) as a salvage therapy for patients with relapsed AML in China. Forty-five patients with relapsed AML were treated with the Mito-FLAG regimen consisting of mitoxantrone (7 mg/m^2^, day 1, 3 and 5), fludarabine (30 mg/m^2^, days 1–5), Ara-C (1 g/m^2^, over 3 h every 12 h, days 1–5) and G-CSF [5 *μ*g/kg/day subcutaneously from day 0 until the white blood count (WBC) was >20×10^9^/l]. Patients with a partial response (PR) received another course of the same regimen. Patients with a suitable donor and aged <50 years received allogeneic stem cell transplantation (allo-SCT). Twenty-three patients (51%) and 3 patients (7%) achieved complete remission (CR) and PR, respectively, following one or two courses of Mito-FLAG, and the overall response (OR) rate was 58%. Nine patients (20%) received allo-SCT and 4 patients (9%) succumbed early. Hematological toxicity and infections were the most prominent toxicities of this regimen. Other toxicities included nausea, vomiting, bleeding, hyperbilirubinemia, renal toxicity and arrhythmia. The probability of overall survival (OS) at 4 years was 19% (95% CI, 11–26%) and the probability of 4-year disease-free survival (DFS) was 29% for all 23 patients in CR (95% CI, 18–41%). Our data suggest that Mito-FLAG is a highly effective and well-tolerated salvage regimen for relapsed AML.

## Introduction

Despite the achievement of an initial complete remission (CR) in the majority of patients with acute myeloid leukemia (AML), the therapeutic results at present remain unsatisfactory. With one or two courses of standard first-line induction chemotherapy, 60–80% of patients with newly diagnosed AML achieve CR; however, 50–70% of them eventually relapse. The disease-free survival (DFS) rate and the overall survival (OS) rate remain low (1–2). Currently, there are no standard salvage regimens for relapsed AML; therefore, alternative treatments that produce improved outcomes for these patients should be explored.

Cytosine arabinoside (Ara-C) remains one of the most effective drugs in AML therapy (3). Fludarabine, a purine analog, markedly increases the intracellular accumulation of Ara-C triphosphate (Ara-CTP), the active metabolite of Ara-C (4). The combination of a middle dose of Ara-C along with fludarabine has also achieved a satisfactory CR rate in refractory or relapsed AML, from 47.5 to 64% (5,6). Adding another chemotherapeutic agent to the FLAG regimen has become one of the hot issues of study. A number of studies have reported on FLAG with mitoxantrone for treating patients with refractory or relapsed AML. The CR rates were 59 and 67%, respectively (7,8). However, to date there have been no reports of this regimen for relapsed AML solely. Since 2003, we have used fludarabine combined with a middle dose of Ara-C, mitoxantrone and granulocyte-colony stimulating factor (G-CSF) as a salvage regimen for patients with relapsed AML in China.

## Patients and methods

### Patients

Between December 2003 and March 2008, 45 patients (22 males, 23 females, aged 17–61 years, median 34 years) with relapsed AML were treated with the Mito-FLAG regimen after obtaining informed consent. These patients had received standard first-line induction chemotherapy following initial diagnosis. Hematologic relapse was defined by recurrence of >5% blasts in the bone marrow (BM). Diagnosis and classification of AML were performed according to the French-American-British (FAB) Corporation criteria (9). The clinical features of the patients are listed in Table I. The study was approved by the ethics committee of Wenzhou Medical College (Wenzhou, China).

### Methods

The Mito-FLAG regimen consisted of five days of treatment with mitoxantrone (7 mg/m^2^, days 1, 3 and 5), fludarabine (30 mg/m^2^, days 1–5), Ara-C (1 g/m^2^, over 3 h every 12 h, days 1–5) and G-CSF [5 *μ*g/kg/day subcutaneously from day 0 until the white blood count (WBC) was >20×10^9^/l].

The response criteria were established according to revised recommendations of the International Working Group for Diagnosis, Standardization of Response Criteria, Treatment Outcomes and Reporting Standards for Therapeutic Trials in Acute Myeloid Leukemia (10). CR was defined as <5% BM blasts with a neutrophil count of >1.0×10^9^/l, a platelet count of >100×10^9^/l and no other diseases. Partial response (PR) was established as either 5–25% BM blasts, a ≥50% decrease in BM blasts or <5% BM blasts but with Auer rod presence. No remission (NR) was established for patients who did not fulfill the above criteria. BM aspirate was performed following chemotherapy as soon as the patient achieved peripheral blood morphology values required for CR; however, this was not performed later than 50 days from the start of treatment. Early mortality (EM) was defined as mortality from any cause during the first 30 days from the beginning of the Mito-FLAG therapy.

Patients in PR received another course of the same regimen. Patients in CR received allogeneic stem cell transplantation (allo-SCT) subsequently if aged <50 years with a suitable donor, whereas those without a donor received maintenance therapy. Antibiotic treatment and supportive care were administered according to local guidelines. Patients with treatment failures received another salvage chemotherapy regimen or palliation therapy subsequently. Hematological and extra-hematological toxicity were assessed according to the World Health Organization (WHO) criteria (11).

DFS was calculated from the first day of remission until the evidence of progression. OS was calculated from the start of Mito-FLAG regimen until mortality.

### Statistical analysis

Statistical analysis was performed using the Chi-square test and Kaplan-Meier survival curves were produced. We considered a two-tailed P-value <0.05 to be an indication of statistical significance.

## Results

### Therapeutic effects

Out of the 45 patients, 21 (47%) achieved CR following the first course of Mito-FLAG and 5 (11%) achieved PR. All the patients with PR received second Mito-FLAG regimen and 2 patients achieved CR following the second course. Finally, CR and PR was achieved in 23 (51%) and 3 (7%) patients, respectively, so the overall response rate was 58%. Fifteen patients (33%) were refractory and 4 (9%) succumbed early due to cerebral hemorrhage (n=3) or pulmonary infection (n=1). Patients with abnormal karyotype (n=16, 36%) had a CR rate of 43.8% and patients with early relapse (n=25) and late relapse (n=20) had a CR rate of 40.0 and 65.0%, respectively. However, analysis of corresponding subgroups stratified according to karyotype and early/late relapse did not show significant differences (Table II).

### Toxicity associated with Mito-FLAG regimen

Four patients (9%) succumbed early; three from cerebral hemorrhage (n=3) and one from pulmonary infection. As expected, severe myelosuppression was observed in all patients. Hematological toxicity and infections were the most prominent toxicities of the Mito-FLAG treatment. The median number of days to recovery of neutrophil count (>0.5×10^9^/l) and platelet count (>20×10^9^/l) was 19 days (range, 9–48 days) and 24 days (range, 10–95 days), respectively. Patients received a median of 5 units (range, 0–12 units) of suspended red cells and 5 units (range, 3–23 units) of apheresis platelets. The incidence of serious infections was high. All but 6 patients experienced fever (temperature >38°C) during the neutropenic phase. Twenty-one patients (47%) had confirmed grade III–IV infections (according to the WHO criteria), which included pneumonia (29%), sepsis (11%), enteritis (7%), perianal abscess (4%) and stomatitis (2%). The median duration of antibiotic therapy was 25 days (range, 15–76 days). The other WHO grade III–IV toxicities included nausea and vomiting (22%), bleeding (18%), hyperbilirubinemia (4%), renal toxicity (4%) and arrhythmia (2%). The median time of hospitalization was 46 days (range, 18–108 days).

### Overall survival and disease-free survival

**Figure 1.**
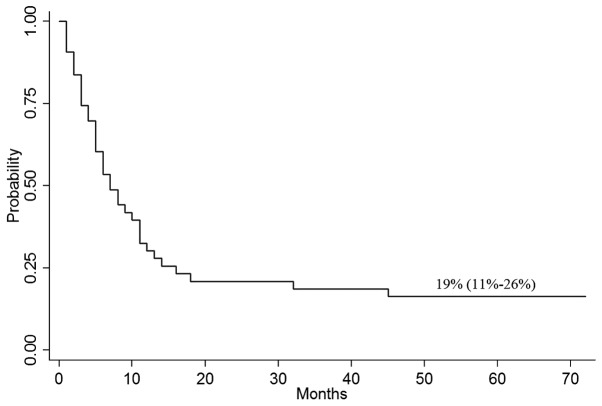
Kaplan-Meier curve for OS in relapsed AML patients treated with Mito-FLAG. OS, overall survival; AML, acute myeloid leukemia.

**Figure 2.**
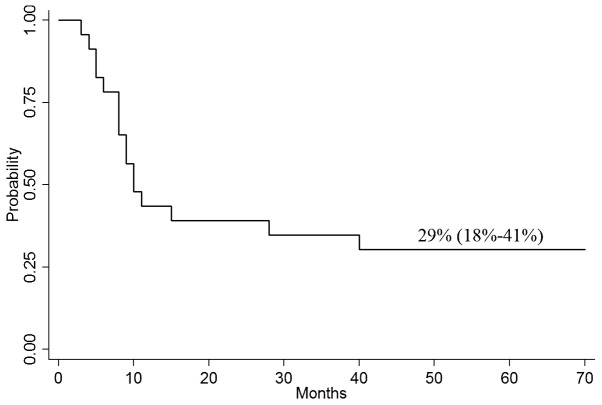
Kaplan-Meier curve for DFS in relapsed AML patients achieving CR. DFS, disease-free survival; AML, acute myeloid leukemia; CR, complete remission.

Two patients were lost to follow-up shortly after receiving induction chemotherapy. The median OS for the other 43 patients was 7 months. With a median follow-up of 11 months (95% CI, 1–72%), the probability of OS at 4 years was 19% (95% CI, 11–26%; Fig. 1). The median time of DFS for all 23 patients in CR was 10 months. The probability of 4-year DFS was 29% (95% CI, 18–41%; Fig. 2).

### Post-transplant outcome

**Figure 3.**
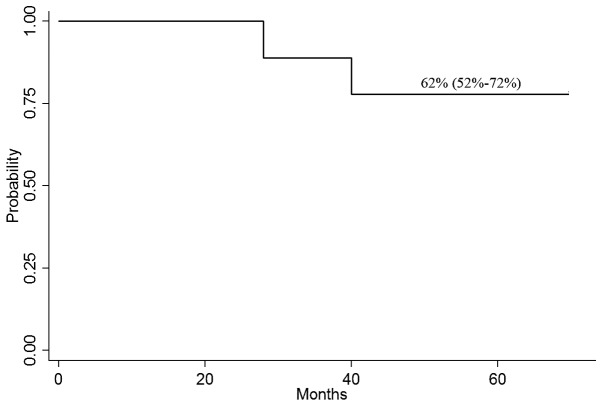
Kaplan-Meier curve for OS in relapsed AML patients undergoing allo-SCT. OS, overall survival; AML, acute myeloid leukemia; allo-SCT, allogeneic stem cell transplantation.

Out of the 23 patients in CR, 9 (20%) received allo-SCT. Among them, 2 patients received allo-SCT with a matched related donor and 7 patients with a matched unrelated donor. Two patients succumbed due to relapse. Seven patients remained in CR. The median OS time for the patients who underwent allo-SCT was 56 months. The probability of OS at 4 years was 62% (95% CI, 52–72%; Fig. 3).

## Discussion

Ara-C is one of the most effective drugs in AML therapy (3). It performs antitumor effects by transforming into 5′-triphosphate Ara-C (Ara-CTP) in cells. A previous study suggested that patients benefit from high-dose therapy when an increase in intracellular Ara-CTP occurs with higher extracellular concentrations of Ara-C (12). Therefore, increased dosages of Ara-C to produce tumor remission rate improvement are often utilized doctors in the clinic. However, the dose escalation of Ara-C did not improve the outcome in practice (13). Thus, the main aim of current research is to determine how to increase the concentration of Ara-CTP in leukemic cells to overcome Ara-C resistance. Fludarabine, a purine analog, which is not susceptible to degradation by adenosine deaminase, maintains *in vivo* activity. By phosphorylation in the plasma, fludarabine transforms to lipophilic 2-F-Ara-ATP which easily passes through the membrane of tumor cells. Following phosphorylation by deoxycytidine kinase, 2-F-Ara-ATP turns to activated F-Ara-ATP, which inhibits the synthesis of DNA, RNA and protein. F-Ara-ATP increases the activity of deoxycytidine kinase, increasing the rate of Ara-CTP accumulation by 2–3 fold (14). Fludarabine, in addition to Ara-C, increases the concentration of Ara-CTP by almost 5 fold in the regimen of Ara-C and G-CSF (15). G-CSF mobilizes quiescent leukemic cells into the cell cycle and increases the sensitivity to Ara-C, the intake of Ara-C and Ara-C-induced apoptosis. G-CSF also enhances the activity of topoisomerase II and accelerates intracellular phosphorylation of fludarabine (16). The FLAG regimen is composed of fludarabine, Ara-C and G-CSF, which has achieved satisfactory CR rate in treating refractory or relapsed AML. The FLAG regimen is repeatedly described as a well-tolerated salvage regimen in AML with CR rates between 47.5 and 68% (5,18,19).

Adding another chemotherapeutic agent to the FLAG regimen has become one of the hot issues of study. A number of studies of FLAG along with idarubicin (IDA-FLAG regimen) for treating patients of refractory or relapsed AML have been reported. The CR rates were 42 and 52%, respectively, which were similar to FLAG (20,21). Mitoxantrone is one of the most common drugs in AML therapy, particularly in refractory or relapsed AML. Further studies indicated that doses of mitoxantrone, a topoisomerase II-directed drug, may be escalated far beyond the conventional dose schedule, with clinical benefit to patients with acute leukemia when combined with Ara-C. The effect is satisfactory and the tolerance is acceptable, even in elderly patients (7,22). FLAG combined with mitoxantrone strengthens the anti-leukemic activity. A number of studies of FLAG along with mitoxantrone for treating patients of refractory or relapsed AML have been reported. The CR rates were 59 and 67%, respectively (7,8).

In our study, the overall response rate was 58%, similar to that of IDA-FLAG. However, the price of idarubicin is more expensive than that of mitoxantrone in China. In addition, idarubicin has been used in the majority of patients with relapsed AML previously and reuse of idarubicin may result in serious cardiac toxicity and other toxicities. Therefore, Mito-FLAG was selected for relapsed AML in our study. Four patients (9%) succumbed early. Although hematological toxicity and infections were the most prominent toxicities of the Mito-FLAG treatment, the patients recovered quickly. The probability of OS at 4 years was 19% and the probability of 4-year DFS was 29% for all 23 patients in CR. Nine out of 23 patients in CR received allo-SCT and 7 of them remained in CR at the end of follow-up. These results indicate that the Mito-FLAG regimen is a highly effective and well-tolerated salvage regimen in relapsed AML. The toxicity is acceptable, enabling the majority of patients to receive further treatment including allo-SCT.

## Figures and Tables

**Table I.** Clinical features of the patients by subtype of AML (M1-6).

Characteristics	All	M1	M2	M4	M5	M6
Male/female	22/23	0/1	8/11	10/7	3/4	1/0
Age (years)						
Median	34	48	32	36	28	30
Range	17–61	-	18–61	22–60	17–50	-
Early/late relapse	25/20	1/0	10/9	8/9	5/2	1/0
Duration of prior remission (months)						
Median	10	4	11	10	9	3
Range	3–50	4	4–50	5–43	6–15	3
Karyotype						
Normal	29	1	14	11	3	-
Abnormal^a^	16	-	5	6	4	1
Number of prior regimens						
Median	3	1	3	3	3	1
Range	1–6	1	1–6	2–6	2–4	1
Number of prior courses						
Median	6	3	6	7	6	2
Range	2–17	3	3–17	4–16	4–9	2
Number of prior chemotherapeutics						
Idarubicin	37	1	15	14	6	1
Daunomycin	13	-	5	6	2	-
Mitoxantrone	17	-	7	7	3	-
Aclacinomycin	22	-	12	8	2	-
Homoharringtonine	14	-	6	6	2	-
Etoposide	14	-	7	5	2	-
Ara-C (monotherapy)	29	-	13	12	4	-

a The abnormal karyotype included +8, −7, 20q^−^, 8q^−^ and complex. No patient had t(15;17), t(8;21) or inv(16). Ara-C, cytosine arabinoside.

**Table II.** Outcome of patients treated with Mito-FLAG.

	CR	%	P-value
Karyotype			
Normal	16/29	55.2	0.463
Abnormal	7/16	43.8	
Relapse			
Early	10/25	40.0	0.095
Late	13/20	65.0	

Data were assessed with univariate analysis (Chi-square test). CR, complete remission.
